# Anxiety state impact on recovery of runners with lower extremity injuries

**DOI:** 10.1371/journal.pone.0278444

**Published:** 2022-12-01

**Authors:** Aimee Madsen, Sharareh Sharififar, Jordan Oberhaus, Kevin R. Vincent, Heather K. Vincent

**Affiliations:** Department of Physical Medicine and Rehabilitation, College of Medicine, University of Florida, Gainesville, Florida, United States of America; Anglia Ruskin University, UNITED KINGDOM

## Abstract

This prospective cohort study examined the impact of high anxiety levels on psychological state and gait performance during recovery in runners with lower body injuries. Recreational runners diagnosed with lower body injuries who had reduced running volume (N = 41) were stratified into groups using State Trait Anxiety Inventory (STAI) scores: high anxiety (H-Anx; STAI ≥40 points) and low anxiety (L-Anx; STAI <40 points). Runners were followed through rehabilitation to return-to-run using monthly surveys. Main outcome measures included kinesiophobia (Tampa Scale of Kinesiophobia, TSK-11), Positive and Negative Affect Schedule (PANAS; Positive and negative scores), Lower Extremity Function Scale (LEFS), running recovery (University of Wisconsin Running Injury and Recovery Index [UWRI]) and CDC Healthy Days modules for general health, days of anxiety/tension, disrupted sleep and work/usual activities. Running biomechanics were assessed at baseline and the final visit using 3D motion capture and a force-plated treadmill. The time to return-to-running for was 5.0±3.1 and 7.9±4.1 months for L-Anx and H-Anx, respectively and participants who withdrew (n = 15) did so at 7.7±6.2 months. L-Anx maintained low anxiety and H-Anx reduced anxiety from baseline to final visit (STAI = 31.5 to 28.4 points, 50.4 to 37.8 points, respectively), whereas the withdrawn runners remained clinically anxious at their final survey (41.5 to 40.3 points; p < .05). Group by time interactions were found for PANAS positive, LEFS UWRI, general health scores, and days feeling worry, tension and anxiety (all p < .05). Final running performance in L-Anx compared to H-Anx was most improved with cadence (8.6% vs 3.5%; p = .044), impact loading rate [-1.9% vs +8.9%] and lower body stiffness [+14.1% vs +3.2%; all p < .05). High anxiety may identify runners who will experience a longer recovery process, health-related functional disruptions, and less optimization of gait biomechanics during rehabilitation after a lower extremity injury.

## Introduction

More than 40 million people participate in running each year in the U.S [1]. Recreational runners vary widely in demography, experience and running technique [2]. While running confers numerous health benefits on physical and psychological well-being, between 20% to 80% of runners may be managing a musculoskeletal injury at any given time [3, 4]. Forty percent of running-related injuries are overuse injuries and 30% are related to previous injuries [5]. Among recreational runners, injuries occur at a rate of 7.7 per 1000 running hours and rates can be as high as 17.8 injuries per 1000 hours of running in novices [1]. Running-related injuries most frequently occur in the lower body. The most common site of a running-related injury in females is the knee, and among males, injuries tend to be distributed across the knee, shank, ankle and foot [6]. Overuse injuries are more common than acute injuries [4]. Patellofemoral pain syndrome, Achilles tendinopathy and medial tibial stress syndrome are among the most commonly-reported lower body injuries in runners with prevalence ranging from 8% to 20% [6, 7].

Recovering from a physical injury may require refraining from running temporarily and can involve various medical management approaches depending on the type and severity of the injury. Soft tissue injuries may be managed with therapy with minimal running time lost (e.g., patellofemoral pain), whereas critical stress fractures (e.g., femoral neck fracture) may require significant time away from running therapy and gradual return-to-run during a six-month period (summarized in [8]). Withdrawal from participation in running and other sports can cause mental stress, such as anxiety, depression and mood disturbance [9–11]. Anxiety can also be increased after an injury [12], and this condition is associated with fear of reinjury. Emotional responses to injury include sadness, anger, frustration, sleep disturbance and disengagement [11]. Short-term deprivation of exercise from as short as one day to two weeks can elevate anger, fatigue, confusion, and lower vigor [13]. Long-term deprivation persisting for months can also be associated with elevated incidence of fear avoidance and less positive affect/ coping [14]. Psychological distress is generally elevated after injury, but improves over time. Emotional reactions to injuries are normal in athletes, but negative reactions that worsen or do not resolve over time can interfere with overall healing and wellbeing [15, 16].

Anxiety can interfere with rehabilitation progress in athletes [17]. Cross sectional studies in adolescent and adult runners show that running-related injury is also related to wellbeing and various QOL indicators like sleep and days without musculoskeletal pain [18, 19]. Despite these emerging relationships, the current clinical paradigm for running injury still primarily focuses on physical treatment. What remains unknown is how specific running-related QOL and indicators, such as sleep and days impacted by pain, change during the recovery process depending on anxiety level. Moreover, it is not clear how anxiety may modify psychological well-being and improvements in physical running performance during rehabilitation. A comprehensive understanding of recovery of runners from both the physical and psychological perspectives will help care teams improve rehabilitation program content and protocols.

Therefore, the purpose of this study was to examine the effect of anxiety on subjective and objective performance measures during the recovery process from a lower extremity running-related injury in recreational runners. We hypothesized that: 1) compared to low anxiety level, high anxiety would correspond to worse mood state, kinesiophobia, running-related QOL and QOL indicators (days of uninterrupted sleep, days disrupted by pain), and 2) high anxiety would correspond to less improvement in specific biomechanical features of running motion.

## Methods

### Study design

This was a prospective, observational study of the psychological wellbeing and gait performance of a cohort of runners who were seeking rehabilitation treatment for lower extremity injury. This study and its procedures followed the guidelines for the Declaration of Helsinki’s protection of Human Subjects. The study and its procedures were approved by the University of Florida’s Institutional Review Board (study number 201700745).

### Participants

Recreational runners were sequentially recruited and followed during the time frame of November 2017-June 2020 in the UF Health Running Medicine Clinic. Study participants included: men and women aged 18–65 years; seeking rehabilitation care and treatment for current running-related injury that had prevented running or caused them to decrease their current regular running volume (Injury has been commonly defined as presence of pain, or decreased ability to run for at least one week [1]); free of symptomatic cardiovascular disease. Runners were excluded if they were using medications that impaired gait or balance. They were also excluded if they were unable to understand the procedures of the study, or if they were pregnant. All participants provided written informed consent. A total of 60 potential participants inquired and underwent an initial screening, and a total of 41 met the inclusion/exclusion criteria and agreed to participate.

### Study schedule

Participants attended a baseline visit after they received the diagnosis of the injury from the physician and had been prescribed physical therapy rehabilitation. After the informed consent process, survey data and gait analyses were performed at this baseline visit. Survey data were then collected monthly using a REDCap online platform [20] until the runner indicated that they had returned back to full running volume. The participants then completed a final set of surveys and a follow-up gait analysis at the final visit. Survey-based findings are reported for each participant as initial visit, midway during recovery and at full recovery.

### Surveys of psychological wellbeing and perceived functional ability

Several measures of psychological distress, mood states were administered at each time point described above. Perceived functional ability for general lower extremity function and running-specific domains were administered using two surveys.

#### State-Trait Anxiety Inventory (STAI)

The state version of the Spielberger’s STAI is valid and reliable measure the transient levels of anxiety. The STAI item inventory is a unidimensional tool for assessing anxiety, with an individual’s state-anxiety score calculated by totaling scores on differing questions assessing several components of anxiety including apprehension, tension, nervousness, and worry. The inventory is comprised of 20 questions scored on a 4-point Likert scale (1 [not at all] to 4 [very much so]) [21]. The STAI has been previously used in recreational runners and marathoners [22, 23]. Based on cutoffs for probable clinical anxiety (>40 Points) [24], participants were stratified into low anxiety (L-Anx; STAI <40 points) and high anxiety (H-Anx; ≥40 points) groups.

#### Positive and Negative Affect Schedule (PANAS)

The PANAS is a 20-question self-report measure quantifying positive and negative affect on a 5- point Likert scale [25]. The 10 positive affect items are: interested, excited, strong, enthusiastic, proud, alert, inspired, determined, attentive, and active. The 10 negative affect items are: distressed, upset, guilty, scared, hostile, irritable, ashamed, nervous, jittery, and afraid. The test-retest reliability of the positive affect and negative affect scales are 0.89 and 0.85, respectively [25]. The PANAS positive affect scores are sensitive to changes in physical activity level [26].

#### Profile of Mood States (POMS)

Studies have reported relationships between exercise and mood, and that there are chronic mood changes with clinical populations [27]. The POMS is a widely used instrument in physical activity research [28]. The instrument contains 65-items and respondent rate each item using a Likert scale with anchors ranging from “*Not at all*” to “*Extremely*”. Items can be combined to form six separate subscales: tension-anxiety, depression-dejection, anger-hostility, vigor-activity, fatigue-inertia and confusion-bewilderment. This instrument has been administered to runners and responds to improvements in running performance [29].

#### CDC healthy days core module

To determine how specific aspects of the QOL change with the time course of recovery from injury, the Centers for Disease Control (CDC) Health-Related Quality of Life (HRQOL-4) survey was administered. This It is a valid [30] and reliable [31] instrument that contains global assessment of health related QOL, unhealthy days and mental health. In this analysis, specific items from this survey were included: ‘general health’, number of days in the last month the participant felt ‘tense/worried’, ‘anxious’, ‘very healthy and full of energy’, number of days in the last month that were ‘disrupted in sleep’ and ‘disrupted in performing usual activities and work’. The mental health days has also been used dichotomously where 14 or more poor mental health days represents “frequent mental distress” [32]. Using this survey, performing exercise is related to fewer mentally and physically unhealthy days [33].

#### Tampa Scale of Kinesiophobia (TSK-11)

The TSK will be used to measure fear of movement or reinjury in patients with pain. The modified version of the TSK (composed of 11 questions, TSK-11) was used in this study because of the invariant nature of the instrument across conditions and patient populations. Each item was provided with a 4-point Likert scale with scoring alternatives ranging from “strongly disagree” to “strongly agree” [34]. This instrument is characterized by two lower-order factors (somatic focus and activity avoidance focus) [35]. The somatic focus represents the beliefs of underlying and serious medical problems, and the activity avoidance focus represents the belief that participation in activity could result in (re)injury or increased pain.

#### University of Wisconsin Running Injury and Recovery Index (UWRI)

This is 9-item instrument designed by the University of Wisconsin faculty to determine longitudinal change in self-rated running ability after running-related injury [36]. Internal consistency is F061 = 0.82 and the test-retest reliability coefficient is 0.93. Likert style choices are provided for questions relating to how the injury is affecting confidence with running, achieving longest weekly distance, longest run distance, pain with running, frustration level and ability to perform daily activities. This measure is responsive to changes in running-related function after an injury.

#### Lower Extremity Functional Scale (LEFS)

The LEFS is a widely-used instrument to detect the effects of lower extremity injuries on perceived physical function difficulties [37]. It is a reliable, valid and responsive tool for different lower extremity conditions [38]. The LEFS consists of 20 question items that are rated in a 0–5 point scale, where higher scores represent better functional status. The total LEFS point range is from 0–80 points. The pooled estimate of the minimal detectable change at the 90% confidence level varies between six and nine points, which are also indicative of clinically meaningful change [38]. This instrument has previously been used in runners with chronic overuse injuries like iliotibial band syndrome [39].

### Gait performance

Gait performance was measured at the beginning of the rehabilitation process and at the completion of rehabilitation when the runner had returned to running. For participants who withdrew or were lost to follow-up, only the baseline test was available.

#### Temporal spatial and kinetic parameters

**Fig 1:**
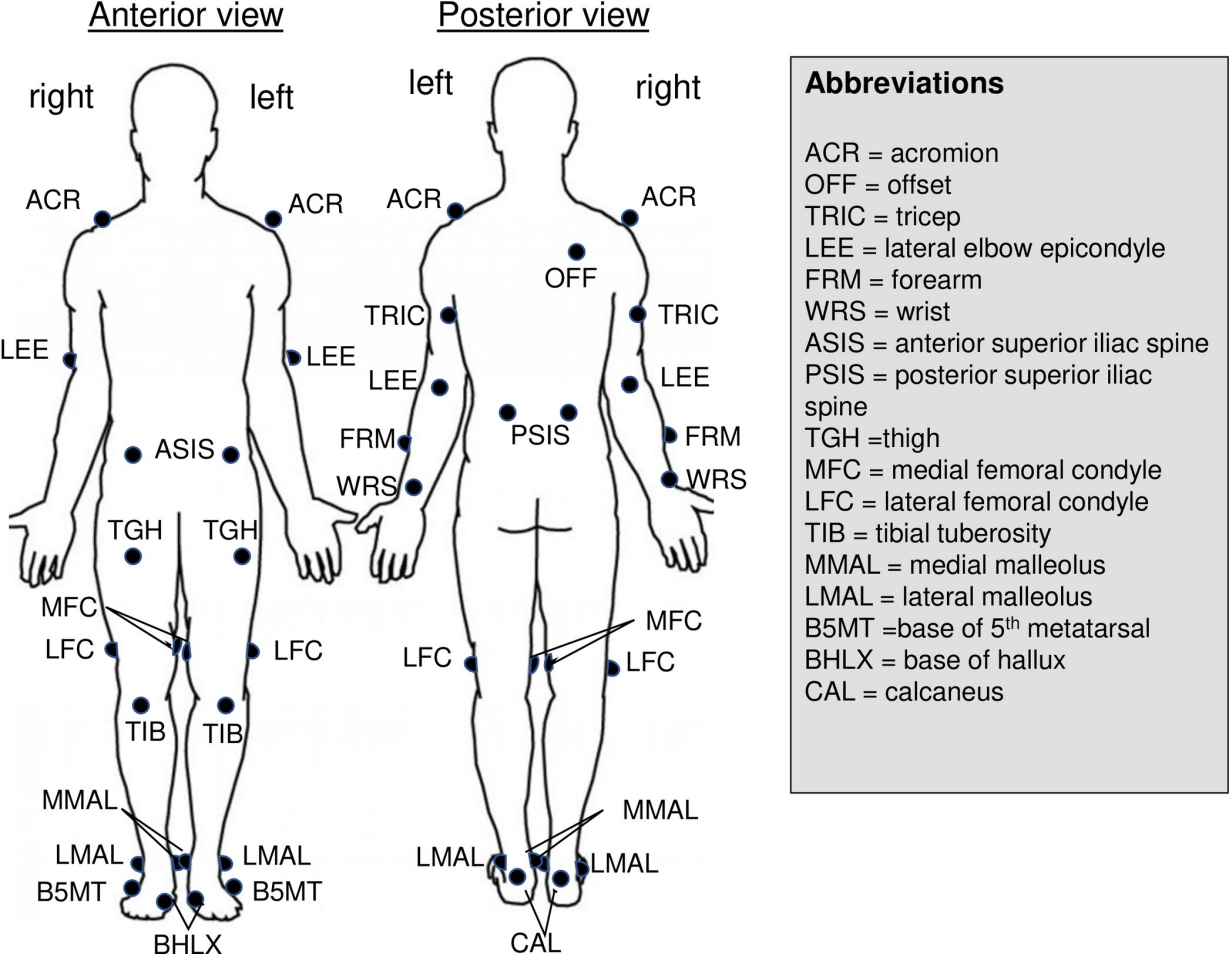
Anatomical placement of retroreflective markers for running tests.

The capture of gait performance at the initiation of rehabilitation and at full return to running allowed the investigators to examine functional changes relative to the psychological changes in runners with different anxiety levels. After running for 10 minutes to establish consistent biomechanical patterns, data were captured. Kinematic and kinetic data were collected using a seven-camera three-dimensional motion capture system (Motion Analysis,) at 120 Hz while participants ran at self-selected speed at 0 grade. Ground reaction forces were collected at 1200 Hz using an instrumented treadmill (AMTI Inc., Watertown, MA) synchronized to the motion capture system. Thirty-three retro-reflective markers were secured to track movement of the body segments from the shoulder to the foot (See Fig 1 for placement of the markers specific landmarks) [40]. A standing calibration trial was recorded prior to data collection. The kinematic data were processed using Visual 3D software (C-Motion Inc., Rockville, MD). Cadence, step length, vertical displacement of the center of gravity, step width, and stance/swing times were calculated as we previously described [41] from data that were averaged from 10 consecutive strides [42].

Bone models were developed for each runner with the individual center of gravity (COG) location. The COG was calculated using the method described by de Leva [43]. Gait cycle time was expressed in percent, where 0% was the initial foot contact and 100% was the foot contact of the same foot after swing phase. Standard temporal spatial parameters included cadence (steps/min), the vertical displacement of the center of gravity (or COG; calculated as the difference in the maximal and minimal vertical height of the estimated COG during an average gait cycle). The distance between two sequential placements of the same foot was the stride length; a stride was comprised of two step lengths (right and left), and is the distance at which the named foot moved forward in front of the other. The step length was calculated as the distance in the walking direction between the proximal end position of the contralateral foot at the previous contralateral foot strike to the proximal end position of the ipsilateral foot at the ipsilateral foot strike plus the distance traveled by the treadmill belt under the stance foot. Stride width was calculated as the medial-lateral distance between proximal-end position of the foot at ipsilateral foot strike to the proximal-end position of the foot at the next contralateral foot strike. The stance time was determined as the time that each foot was in contact with the treadmill belt, and was expressed relative to the gait cycle. The swing time was determined from the remaining time of the gait cycle not in stance. The medial-lateral range of motion excursion of the COG was calculated as the maximal difference between the medial-lateral positions of the estimated COG during an average gait cycle. Footstrike type was determined by the angle between the foot segment and the horizontal ground at foot contact.

Three kinetic measures represented loading, including the vertical peak ground reaction force (GRF) normalized to body weight, the vertical average loading rate (VALR) normalized to body weight. A threshold of 100 N in GRF was established to identify initial foot contact and toe-off. GRF data were filtered at 9Hz using a fourth order low-pass Butterworth filter with a cutoff frequency of 40 Hz and normalized by body weight. VALR was calculated from ΔF/Δt from the initial linear portion of the force curve using the techniques described by Samaan et al. [44]. In cases where GRF curves had an initial impact peak, the ΔF in vertical GRF from 20%–80% of the initial peak was calculated [42]. Vertical stiffness was estimated using the following calculation:

$${\text{K}}_{\text{vert}}={\text{F}}_{\text{max}}/\Delta \text{y}$$

where F_max_ is the peak vertical force and Δy is the maximum displacement of the COG [45]. Right and left side values for each of these measures were averaged and reported in the results.

### Statistical analysis

All deidentified data available in S1 Appendix that support Tables 1–3. Statistical analyses were performed using SPSS version 28.0 (IBM, Chicago, IL). Normality of the data was confirmed using Shapiro-Wilk tests, and descriptive statistics were calculated on all study variables and demographics. One-way analysis of variance (ANOVA) were performed to determine if continuous baseline variables were different between L-Anx and H-Anx groups. Chi square tests were used to determine if there were differences in categorical variables (injury location distribution, pain scores and withdrawals) between groups. Repeated measures ANOVA were conducted to test the interaction of group by time for psychological survey and QOL outcomes; the between group factor was grouping by anxiety level and completion (L-Anx, H-Anx, Withdrawm/did not complete the study) and the within group factor was time point (baseline, midway, final time point); age, BMI and months of recovery were covariates. Repeated measures ANOVA were also used to test group by time interactions for gait performance variables, where the between group factor wan anxiety level and completion (L-Anx, H-Anx, Withdrawm/did not complete the study) and the within group factor was time point (baseline, final time point) using age, BMI and months of recovery as covariates. Effect sizes between completers in the H-Anx and L-Anx groups were determined for key outcomes using the Cohen’s *d* tests, where values were classified as small (0.20), medium (0.50) and large (0.80) [46].

**Table 1:** Baseline characteristics in injured runners with and without high anxiety levels.

	High Anxiety (n = 18)	Low Anxiety (n = 23)	p
**Female Sex (#, %)**	16 (88.9)	19 (82.6)	.577
**Age (yr)**	35.7 ± 11.6	32.6 ± 10.4	.382
**Height (cm)**	163.8 ± 7.9	168.5 ± 8.5	.086
**Weight (kg)**	61.4 ± 7.0	61.4 ± 8.7	.994
**BMI (kg/m** ^**2**^ **)**	22.9 ± 2.6	21.6 ± 2.1	.086
**Long run distance (km)**	12.1 ± 7.2	11.3 ± 7.2	.742
**Runs per week (#)**	4.2 ± 1.1	3.7 ± 1.8	.330
**Weekly distance (km)**	27.4 ± 25.4	29.8 ± 25.9	.772
**Months in study (#)**	8.3 ± 5.9	5.6 ± 3.5	.070
**Taking anti-anxiety meds (#, %)**	4 (22.2)	3 (13.0)	.358
**STAI State score (points)**	50.4 ± 7.1	31.5 ± 5.0	< .0001
**STAI Trait score (points)**	48.3 ± 11.5	34.1 ± 8.7	< .0001
**Injury locations (#)**			
**Low back/pelvis**	2	6	.111
**Hip**	7	6	.382
**Knee**	4	10	.154
**Lower leg**	4	3	.438
**Ankle**	2	2	.796
**Foot**	5	10	.300
**Injury type (# sites)**			
**Stress fractures**	8	4	.071
**Non-bony injuries**	18	29	.180
**Current average weekly pain at injury site (NRSpain, points)**	3.3 ± 2.2	3.0 ± 2.4	.605
Withdrew* (#, %)	8 (44.4)	8 (34.8)	.529

*Withdrawn or lost to follow-up, did not return to regular running

BMI = body mass index; STAI = State Trait Anxiety Inventory; NRSpain = numerical pain rating scale

Values are means SD or % of the group.

Note: some runners had histories both stress fracture and non-fracture injuries.

**Table 2:** Psychological well-being in injured runners with and without high anxiety levels and runners who withdrew (WD). Values are means (SD).

	H-Anx (n = 10)			L-Anx (n = 15)			WD (n = 15)						
	Baseline	Midway	Final	Baseline	Midway	Final	Baseline	Midway	Final	Group	Time	Interaction	Cohen *d*
**STAI State (pts)**	50.4 (8.6)	47.3 (12.5)	37.8 (8.1)	31.4 (5.4)	30.3 (6.7)	28.4 (8.1)	41.5 (11.0)	42.1 (10.5)	40.3 (8.9)	< .001	.288	.317	1.40
**TSK-11 (pts)**	25.3 (5.2)	23.1 (4.6)	19.9 (3.9)	21.6 (3.2)	18.5 (3.9)	16.7 (4.0)	24.9 (6.0)	22.1 (5.3)	24.4 (6.8)	.016	.028	.081	0.30
**PANAS positive (pts)**	27.9 (7.2)	28.1 (6.6)	33.8 (8.2)	36.1 (5.8)	39.1 (8.0)	38.9 (8.4)	31.5 (5.6)	28.7 (6.6)	29.1 (5.6)	.736	.002	.032	0.42
**PANAS negative (pts)**	24.6 (7.7)	23.1 (6.1)	17.2 (4.6)	14.4 (3.7)	14.6 (3.4)	14.2 (5.1)	18.5 (5.7)	17.9 (5.1)	17.7 (6.4)	< .001	.088	.177	0.94
**^LEFS (pts)**	60.5 (18.9)	73.0 (5.7)	77.0 (3.3)	66.5 (10.9)	75.5 (4.2)	78.6 (2.6)	64.7 (8.2)	66.1 (6.6)	64.3 (10.2)	.006	.003	.008	0.48
**UWRI (pts)**	14.7 (5.1)	19.1 (6.6)	29.2 (5.5)	13.7 (5.1)	23.2 (4.2)	28.7 (3.0)	11.1 (4.6)	13.9 (6.6)	13.6 (8.8)	< .001	< .001	< .0001	0.11
**CDC Healthy Days Core Module, specific items scored 1 to 5 points (where 1 = excellent, 3 = good, 5 = poor)**													
**General health (pts)**	1.9 (0.8)	2.2 (0.8)	1.6 (0.5)	1.4 (0.6)	1.4 (0.6)	1.5 (0.6)	1.8 (0.8)	2.3 (0.9)	2.3 (0.9)	.046	.168	.037	0.32
**CDC Healthy Days Core Module, days in last month the participant felt:**													
**Tense, worried anxious (d)**	20.5 (17.1)	11.5 (2.5)	4.5 (2.5)	8.3 (10.4)	7.8 (7.9)	5.1 (7.6)	9.4 (8.6)	11.9 (11.3)	7.9 (6.7)	.135	.062	.041	1.36
**Very healthy and full of energy (d)**	10.6 (7.2)	14.9 (8.0)	21.3 (5.7)	19.8 (6.7)	19.7 (8.4)	21.0 (8.5)	12.8 (6.7)	11.9 (10.0)	11.0 (9.4)	.115	.260	.241	1.37
**CDC Healthy Days Core Module, days in last month participant experienced disruption to:**													
**Sleep (d)**	15.0 (6.5)	9.2 (9.0)	7.0 (4.0)	8.4 (5.8)	8.7 (9.8)	5.2 (5.1)	12.5 (9.1)	14.8 (9.0)	10.5 (5.2)	.131	.817	.850	0.88
**Usual activities, work due to pain (d)**	10.3 (11.2)	3.3 (4.9)	0.3 (0.7)	12.2 (12.0)	1.1 (2.0)	0.0 (0.0)	8.3 (10.3)	2.2 (3.8)	4.0 (9.9)	.960	.751	.732	0.37

Note: One participant in the withdrawn group did not fully complete the surveys and was not included in the analysis. Models are adjusted for age, BMI and months in study.

^ skewed and kurtosis

TSK = Tampa Scale of Kinesiophobia; PANAS = Positive and Negative Affect Schedule; LEFS = Lower Extremity Functional Scale; UWRI = University of Wisconsin Running Injury and Recovery Index

**Table 3:** Performance-based gait metrics in injured runners with and without high anxiety levels. Values are means (SD).

	H-Anx		L-Anx		WD				
	Baseline	Final	Baseline	Final	Baseline	group	time	interaction	Cohen’s *d*
**Cadence (steps/min)**	168 (7)	174 (6)	163 (9)	177 (10)	169 (10)	.531	.044	.044	0.98
**COG vertical displacement (cm)**	8.9 (1.9)	8.7 (1.2)	10.0 (1.4)	8.9 (1.5)	9.0 (1.5)	.749	.330	.326	0.61
**Step length (m)**	0.91 (0.20)	0.97 (0.14)	0.89 (0.12)	0.97 (0.14)	0.84 (0.12)	.310	.660	.664	0.13
**Stride width (cm)**	9.6 (2.5)	9.3 (2.6)	10.6 (4.0)	10.5 (3.8)	8.0 (2.3)	.447	.910	.905	0.06
**Stance time (% GC)**	46.2 (2.8)	46.0 (2.4)	46.6 (2.4)	45.6 (1.9)	46.8 (2.0)	.751	.690	.694	0.51
**Swing time (% GC)**	53.7 (2.7)	54.0 (2.4)	53.4 (2.4)	54.4 (1.9)	53.2 (2.0)	.792	.780	.781	0.30
**Peak GRF (BW)**	2.3 (0.4)	2.4 (0.4)	2.3 (0.2)	2.5 (0.3)	2.3 (0.2)	.558	.420	.418	0.30
**Impact loading rate (BW/s)**	65.8 (21.7)	71.7 (17.7)	53.1 (14.0)	52.1 (17.4)	60.9 (20.3)	.018	.990	.996	0.49
**Stiffness (N/cm)**	163.8 (17.7)	169.0 (19.3)	149.7 (25.2)	170.8 (32.0)	151.0 (32.8)	.756	.150	.147	0.66

WD = withdrew or did not complete the study; the data from this group are reported in the table only, but not included in the analysis. Models are adjusted for age, BMI and months in study. COG = center of gravity; GC = gait cycle; GRF = ground reaction force; BW = body weights

## Results

### Participant characteristics

**Fig 2:**
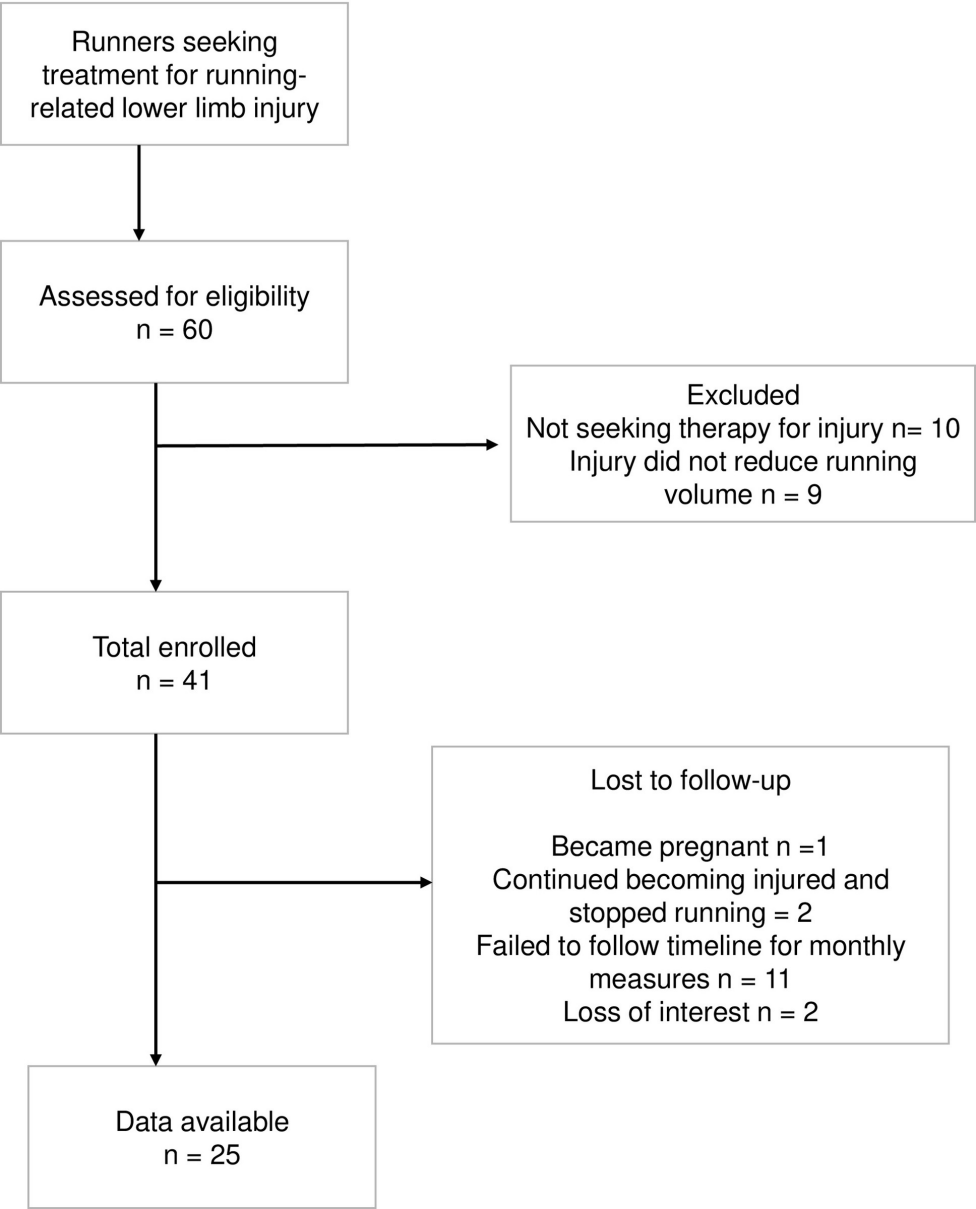
Study flow diagram.

Table 1 compares the baseline characteristics of the H-Anx and L-Anx groups. Groups are well-matched with respect to anthropometrics, location and type of injury and the number of withdrawals. Stress fractures occurred at the tibia (n = 5), metatarsal (n = 6) femoral neck (n = 1). Non-bony tissue running-related injuries included patellofemoral pain (n = 8), hip labrum pain (n = 4), various tendinitis (peroneal, flexor hallicus longus, patellar, iliotibial band), ligament inflammation and knee meniscal injury (n = 35) Typical weekly training distances and running session frequency were also similar. The H-Anx had a longer recovery duration in the study (p = .035). As expected, the H-Anx group reported 60.0% and 41.6% higher scores on the STAI in both trait and state categories, respectively, compared to the L-Anx (p < .0001). A total of eight participants per group withdrew after a mean participation time of 7.6 months in the study. The mean age and anthropometrics and running were not different in the participants who withdrew compared to the H-Anx and L-Anx groups. The average months of participation in the withdrawn group was 7.7±6.2 months, compared to the completers in the L-Anx and H-Anx groups who participated and average of 5.1±3.1 and 7.9±4.1 months, respectively (p = .233). Differences in injury location or type were not detected among the L-Anx, H-Anx and participants who were withdrawn. Fig 2 provides the study flow diagram with reasons for withdrawal.

### Psychological well being survey outcomes

Table 2 shows the prospective outcomes for the L-Anx, H-Anx and participants who did not complete the study and were withdrawn. Significant group by time interactions were detected for PANAS positive scores, LEFS scores and UWRI scores (all p < .05). Greater changes over time in the H-Anx for PANAS positive and LEFS and in the L-Anx for UWRI scores. In addition, significant interactions were found for the CDC Healthy Core Module ratings of General health, and the number of days feeling tense/worried and anxious, where the participants who did not complete the study demonstrated worse scores by the final time point than the other two groups (p < .05). Main effects were shown for group with respect to STAI state scores and TSK-11 scores, with the withdrawn group demonstrating consistently high anxiety scores and kinesiophobia by the final time point (p < .001). Also, the H-Anx group maintained a higher PANAS negative level, lower General Health scores, lower UWRI scores and reported more days disrupted in usual activities compared to the L-Anx over time (all p < .05). Effects sizes on anxiety level for the participants who completed the study on outcome change scores ranged from small to large (*d* range = 0.11 to 1.40).

### Running biomechanical changes from baseline to recovery

Runners who completed the study successfully returned to pre-injury running volumes. Habitual running speed increased from 9.4 ± 2.7 km/h to 10.6 ± 2.7 in the H-Anx group and from 9.5 ±1.2 km/h to 10.8 ± 1.4 km/h in the L-Anx group from the initial to final testing visit. Table 3 provides the performance-based gait metrics at baseline and the final visit. The interaction for cadence was significant (p = .044), with the L-Anx demonstrating greater increases in cadence values over time compared to H-Anx. Both groups improved running speed, stance, and swing times during recovery. The H-Anx group two demonstrated higher impact loading rates at each time point compared to L-Anx (p = .018). Change scores (difference from baseline to final time point) in stiffness were significantly greater in the L-Anx group compared to the H-Anx group (21.0 ± 19.6 N/cm versus 5.1 ± 12.8 N/cm; p = .034). Effect sizes of anxiety level on the changes in these gait parameters ranged from small to large among the runners who completed the study (*d* range = 0.06 to 0.98).

## Discussion

The effect of anxiety on self-rated and performance-based gait measures in recreational runners during recovery from a lower limb injury was examined. Our hypothesis was that high anxiety levels during rehabilitation would negatively affect mood state, kinesiophobia, running-related QOL and QOL indicators. Our results partially confirm this hypothesis. Both H-Anx and L-Anx groups in general improved in psychological well-being, self-rated performance measures and gait parameters. However, the runners who were withdrawn demonstrated persistent anxiety to the last available follow-up point, higher TSK-11 scores, worse LEFS and UWRI scores with worse select CDC Healthy Days core module scores. All runners improved several gait metrics including speed and cadence upon return to running. However, the H-Anx group maintained high impact loading rates and did not significantly modify leg stiffness at the end of recovery. These findings could be applied to developing rehabilitation targets for runners coping with high anxiety for running-related injuries, such as shifting focus to positive rehabilitation gains and setting specific goals for improving running technique. Importantly, these findings indicate the potential for anxiety and elements of wellbeing (sleep, worry, general health) to be considered for future tracking among runners at the start of rehabilitation.

Directly comparative data in recreational runners are scarce. Lichtenstein et al. [9] found that injured athletes (chronic, acute trauma, combined or unknown) reported higher stress, depression rates and more difficulty with performing exercise, going to work and social activities; 21% indicated they might need psychological help due to persistent negative emotional state. Similar to other work, we found wide variation in time to recover after running related injury from days to months [47]. Among injured runners, Maschke et al. [14] found elevated psychological distress and elevated Athletic Fear Avoidance questionnaire scores. During the 12-16-week recovery, these authors reported an average UWRI score improvement of 61% and fear avoidance score reduction of 9%. Similarly, our H-Anx and L-Anx runners improved UWRI scores 98% and 109%, respectively, suggesting a differential impact of anxiety on these scores. Runners who were withdrawn only increased UWRI scores by 25%. Several studies found that inferior psychological outcome is linked to poorer rehabilitation outcomes after sport injury and treatment, but do not separate the role of mood disorders such as depression from the possible contributions of anxiety [48, 49].

In contrast to our hypotheses, kinesiophobia levels did not significantly change within either group during recovery. Our runners did not demonstrate high TSK scores as has been reported by athletes with other acute injuries like ACL [50]. The existing literature of TSK has focused largely on ACL-related research or among individuals with chronic musculoskeletal pain conditions, both of which are different from the recreational runner population. It is likely that the trauma associated with these running injuries was not substantial enough to provoke fear and kinesiophobia during recovery as would acute severe disabling injuries like ACL rupture or years of painful exposure to degenerative joint disease such as arthritis. However, one study found higher fear-avoidance is associated with a decrease in perceived running ability [14], and other studies have reported persistence in negative mood throughout the recovery process [10]. In our study, the self-reported wellbeing, and the number of days in the last month with feelings of tension, worry and anxiety as reported in the CDC Healthy Days core module improved most in the L-Anx group by the final time point. Given that the QOL survey only documented presence of negative emotion over a number of days and not the severity of the emotion, there is the possibility that L-Anx experienced less intensive negative emotions compared to H-Anx. We did not detect statistical interactions of anxiety level and time on outcomes of days disrupted by sleep and days with difficulty performing usual activities. It is important to note, however, that both groups reported fewer disruptions as recovery progressed, but anxiety and persistent anxiety blunted this benefit over time. Anxiety can negatively impact sleep, and poor sleep is associated with deleterious molecular processes in muscle cells that diminish the body’s ability to recover after muscle damage caused by exercise or injury [51].

Our second hypothesis was that anxiety would correspond to less improvement in specific biomechanical metrics of gait. All runners made improvements in several gait metrics during recovery including speed and several temporal spatial parameters. To date, we are not aware of any reports of anxiety impact on gait biomechanical changes after rehabilitation for lower extremity injury in recreational runners. The H-Anx group demonstrated persistently higher VALR and minimal change in leg stiffness by the final time point. These findings could be clinically-relevant with respect to recurrent injury; generally a history of running injury increases the risk of another injury among recreational runners [52]. Runners who are unable to adjust their technique may be at risk for recurrent, chronic injury. We acknowledge that the role of leg stiffness on injury risk differs among published studies and measurement methods [53–55], and leg stiffness alone may not be a risk factor. Finding the optimal stiffness level that benefits performance and minimizes injury risk [55] will be an important goal as part of rehabilitation. What is unknown is how, compared to other patient factors (e.g., sport identification, socioeconomic status, therapy access [10, 11]), anxiety level modulates adjustability of biomechanical risks during recovery and rehabilitation. We found that throughout recovery, PANAS negative scores remained higher in the H-Anx group, which may be interfering with recovery efficiency and optimization of gait outcomes. Negative mood can also impair perceived athletic ability [14] and produce challenges with readiness to fully engage back into sport [49].

### Clinical applications

Specific steps may be taken to help injured runners move through recovery. First, a shift in mindset toward the positive gains achieved during recovery may help reduce or manage anxiety through specific rehabilitation foci. This may be accomplished through involvement of the athlete as much as possible in local or regional running group meetings or with supportive training partners. For example, while peer runners perform loops/ routes, the injured athlete can participate with walking, or return-to-run programs, pre and post warm-up and strengthening exercise. Second, the care team can help reorient the focus of the runner away from internal to external. Apply techniques that promote positive mental state and rehabilitation adherence including positive self-talk, guided imagery, modeling videos, emotional written disclosure and goal-setting [56]. Clinicians can develop with the patient a tangible set of goals that focus on individual corrections of gait technique (such as those found here, like reducing VALR and controlling leg stiffness) and gains in neuromotor function. Involve the athlete in designing the plan of care as a team after an injury and discuss the rehabilitation process [57]. Consider referrals to mental health professionals if the patient is not progressing and adjusting functionally and continues expression of negative emotions. Third, use of tools to screen for anxiety and depression early in the recovery process may help clinicians identify which runners may have higher likelihood of a challenging or protracted recovery due to anxiety related issues [11, 17].

### Limitations and strengths

For participants whose recovery trajectory was longer than 7 months, retention in the study was difficult. Some of these losses to follow up occurred due to reinjury and most did not comply with monthly surveys by this time point. Possibilities are that completion of monthly surveys was a repeating reminder of the injury impact on QOL and emotional state, or that the monthly surveys were burdensome. However, our dropout falls within the range of 21%-46% of previously reported prospective studies of runners over a period of 2–12 months [58–60]. To reduce the possibility of outcome bias, we included the withdrawn/ drop out in the psychological well-being outcomes, but could not do this for the biomechanical outcomes. Future studies may consider periodic gait assessment to track progress in parallel with psychological measures. Similar to other recent studies [14], we were not able to report the therapy exposure or components for each runner in the study. Therefore, we cannot exclude the possibility of variation in the physical therapy provided, with different emphases and therapeutic goals. Also, social support can play an important role in the rehabilitation process and we did not measure support levels for each runner. This study was observational in nature, and causation of anxiety to changes in any psychological outcome and biomechanical variable cannot be inferred. Given that the health related QOL survey did not detect differences in several domains of wellbeing based on frequency of days with emotional experiences, it is likely that this tool may not be sensitive enough to track outcomes in this population. Additional instruments can be explored specifically for runners. There is the chance that volunteer bias exists in this study, where interested individuals signed up and remained active until completion. Finally, the sample size of this study was relatively small, and the power to detect significance for all outcome variables was limited. Larger cohorts may provide opportunity to detect differences in anxiety effect with different injury histories, sex and age. Strengths of this study include the prospective nature of data collection and inclusion of several self-rated and performance-based measures to capture the whole patient experience with recovery after a running injury. Participants included both men and women, a variety of common running injuries and measurement metrics that are important to daily living QOL and with running. Finally, the effect sizes of the measures included in study can be applied by other researchers in future studies examining anxiety effects on psychological wellbeing and performance in runners.

## Conclusions

High anxiety level may characterize runners who will have greater challenges reducing negative mental affect and fully optimizing running mechanics during recovery from lower body running injuries. Anxiety may also interfere with sleep and daily functional activities among injured runners. During rehabilitation, different techniques may be implemented with the patient to reduce or manage anxiety effects and improve QOL and gait performance outcomes.

## Supporting information

S1 Appendix This is the study data file. This file contains all the data used in this study in tables, figures and analyses.
(XLS)
